# Marginal Accuracy and Internal Fit of 3-D Printing Laser-Sintered Co-Cr Alloy Copings

**DOI:** 10.3390/ma10010093

**Published:** 2017-01-23

**Authors:** Myung-Joo Kim, Yun-Jung Choi, Seong-Kyun Kim, Seong-Joo Heo, Jai-Young Koak

**Affiliations:** Department of Prosthodontics and Dental Research Institute, School of Dentistry, Seoul National University, 101 Daehak-Ro Jongno-Gu, Seoul 03080, Korea; silk1@snu.ac.kr (M.-J.K.); bony0330@hanmail.net (Y.-J.C.); ksy0617@snu.ac.kr (S.-K.K.); heosj@snu.ac.kr (S.-J.H.)

**Keywords:** Co-Cr alloy, CAD/CAM milled, laser sintering, marginal accuracy, internal fit

## Abstract

Laser sintered technology has been introduced for clinical use and can be utilized more widely, accompanied by the digitalization of dentistry and the development of direct oral scanning devices. This study was performed with the aim of comparing the marginal accuracy and internal fit of Co-Cr alloy copings fabricated by casting, CAD/CAM (Computer-aided design/Computer-assisted manufacture) milled, and 3-D laser sintered techniques. A total of 36 Co-Cr alloy crown-copings were fabricated from an implant abutment. The marginal and internal fit were evaluated by measuring the weight of the silicone material, the vertical marginal discrepancy using a microscope, and the internal gap in the sectioned specimens. The data were statistically analyzed by One-way ANOVA (analysis of variance), a Scheffe’s test, and Pearson’s correlation at the significance level of *p* = 0.05, using statistics software. The silicone weight was significantly low in the casting group. The 3-D laser sintered group showed the highest vertical discrepancy, and marginal-, occlusal-, and average- internal gaps (*p* < 0.05). The CAD/CAM milled group revealed a significantly high axial internal gap. There are moderate correlations between the vertical marginal discrepancy and the internal gap variables (*r* = 0.654), except for the silicone weight. In this study, the 3-D laser sintered group achieved clinically acceptable marginal accuracy and internal fit.

## 1. Introduction

Cobalt-chromium (Co-Cr) alloys have been widely used in dentistry for removable partial dentures, metal frames, and porcelain-fused-to-metal crowns, mainly because alloys are strong, resistant to corrosion, and relatively inexpensive, when compared to gold alloys and some all-ceramic materials [1,2,3,4,5]. Base metal alloys may be preferable over noble alloys for implant-retained structures [1,6], due to their higher fracture strength, elastic modulus, hardness, and low cost [7]. However, the fabrication process for alloys is usually difficult because of their high melting point (1349–1449 °C), hardness, and limited ductility [8]. Co-Cr alloy implant superstructures are often associated with marginal and fitting discrepancies. These faults can be attributed to the expansion and contraction of the impression materials, gypsum, wax, investment, and alloy in the lost-wax technique [9]. Lost-wax casting has been the most commonly used method for fabricating dental prosthesis for many decades [1], but errors accumulated in the series of laboratory steps are inevitable. In recent years, there have been attempts to use the conventional casting in combination with the Computer-aided design/Computer-assisted manufacture (CAD/CAM) technology, for milling the fabricated wax pattern, followed by scanning (wax/CAM), instead of the conventional investing and casting procedures [10]. Also, the castable pattern resin has been designed using a three-dimensional system and milled for the fabrication of the copings, in place of the conventional manual wax-up procedures for maintaining the standardized design [11]. The introduction and increased usage of CAD/CAM technology in dentistry has replaced error-prone manual laboratory steps, with aligned industrial manufacturing processes. It can be both time-saving and cost-effective, when compared to conventional casting technology, and many studies have been reported [12,13,14]. Laser sintering is a type of additive manufacturing and a relatively new method compared to both casting and CAD/CAM milling techniques [15,16,17]. This is also called three-dimensional (3-D) printing, or rapid prototyping (RP). Additive manufacturing can directly fabricate 3-D objects from CAD in a single stage, for which X-ray CT and MR images are available [8]. In contrast to CAD/CAM-based cutting technology, additive manufacturing technology creates products layer by layer, on the basis of sliced data from the 3-D design. A laser scans metal powders according to the sliced data, in order to obtain a layer of products. The powders for the next layer are covered with the melted layer, and the laser is again scanned, according to the next sliced data. This sequence continues until the near-net-shape of the product is automatically formed. In addition, free form shaping can be achieved without molds and without the limitations associated with the use of cutting tools. It involves several advantages over the casting and CAD/CAM technique, such as the saving of the raw material and the requirement for fewer tools, which both reduce costs [18]. There have been some studies focusing on a comparison of the mechanical properties and microstructural characteristics of the fractured surfaces of Co-Cr alloys manufactured by casting, CAD/CAM milled, and 3-D laser sintered techniques [19,20,21,22].

Precise marginal and internal fit are two of the most important criteria for ensuring the clinical success of dental restorations. Smaller marginal gaps produce less gingival irritation [23,24] and cement washout [25,26], improving the clinical outcome and longevity of the restoration [27,28,29,30,31]. Subgingival marginal discrepancies in implant-supported restorations are related to changes in the ecologic environment, that may contribute to the occurrence of peri-implantitis or to bone loss in the marginal portion of the implant [24,27].

Although there is no standard method available for measuring the marginal gap, some fit assessment protocols are described in the literature [32,33,34]. One of these is the measurement of the specimens by direct visualization under a microscope. This method is non-destructive and can provide several measuring points, however, it is difficult to obtain accurate measurements and the internal fit cannot be measured. Witkowski et al. used this method in comparison with the marginal fit of the casting and CAD/CAM milled crown-copings [35]. Another method is the measurement of the internal gap in the embedded and sectioned specimens. Alghazzawi et al. compared the marginal adaptation of two types of glass-infiltrated ceramic crown-copings by CAD/CAM technology [36]. Evaluation methods which include impression taking can be divided into the replica technique and the weighting technique. The former, also called the cement analog technique, was initially described by McLean and von Fraunhofer [28]. The latter is the weight measurement of the cement analog layer and recently employed a light-body silicone in place of luting cement to determine relative marginal gaps for ceramic crowns [37]. Nakamura et al. [38] and May et al. [39] used test-fit silicone paste for also measuring the internal gaps. Besides, the clinical evaluation method using the explorer and scoring system, the Micro CT, and 3D analysis, can be used for evaluation of the restorations.

A few published studies on the fit of Co-Cr alloy copings using laser-sintered technology have demonstrated marginal discrepancies on a single tooth crown [15,40,41]. Moreover, there has been little information presented on the marginal and internal gap of Co-Cr alloy copings for single implant restoration. Therefore, a detailed investigation on the direct comparison of the marginal and internal fit of implant supported Co-Cr copings fabricated by laser sintered methods, is needed. The purpose of the present study was to compare the marginal accuracy and internal fit of implant supported Co-Cr copings, fabricated by casting, CAD/CAM milled, and 3-D laser sintered techniques. The null hypothesis is that the fabrication methods have no effect on the marginal accuracy and internal fit of Co-Cr alloy crown-copings.

## 2. Results

The weight of the silicone material ranged from 0.005 g to 0.009 g. The lowest silicone weights were observed in the casting group. There were significant differences in the mean weight between the casting and the CAD/CAM milled groups, but no significant differences were found between the other groups, as shown in Table 1.

The mean two-dimensional vertical marginal gap value is shown in Table 2. The laser sintered copings demonstrated the highest value, while the casting copings significantly exhibited the lowest vertical marginal gap (*p* < 0.003).

**Figure 1:**
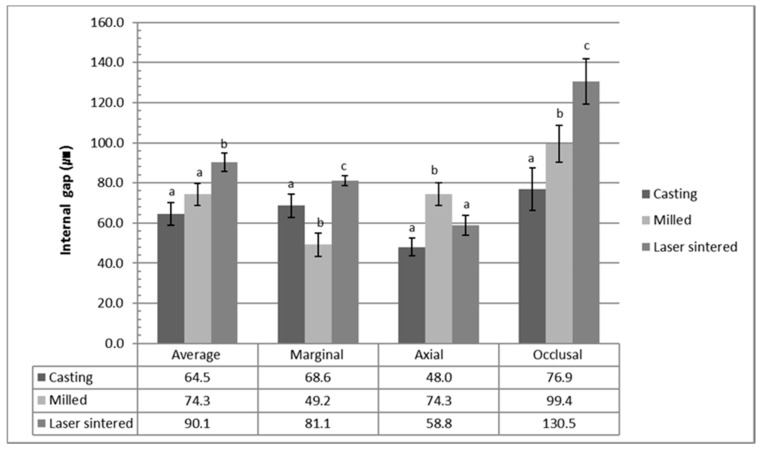
The internal gap in the marginal, axial, and occlusal area for the casting, CAD/CAM milled, and laser sintered copings. The values represent the means and standard deviations (120 points/area of each group). The average internal gap values were calculated by the mean values of the marginal, axial, and occlusal area of each specimen in the group (5 measurements × 2 locations × 3 areas × 12 specimens × 3 groups, total 1080 points). The 3-D laser sintered group showed the highest average internal gap value which is significantly different from those of the casting and the CAD/CAM milled copings (*p* < 0.05). There was no significant difference between the casting and the milled group (*p* > 0.05).

The internal gap of the three groups is shown in Figure 1. The mean average internal gap value was 65.9 ± 11.4 µm in the casting group, 74.3 ± 10.9 µm in the CAD/CAM milled group, and 90.1 ± 9.1 µm in the laser sintered group. The 3-D laser sintered group exhibited the highest average internal gap, which was significantly different to the casting and CAD/CAM milled copings (*p* < 0.0001). There was no significant difference between the casting group and the milled group (*p* = 0.784). The marginal internal gaps were 68.6 ± 11.9 µm, 49.2 ± 11.8 µm, and 81.1 ± 4.9 µm, for the casting, the CAD/CAM milled, and the laser sintered coping, respectively. There were significant differences between all three groups. On the other hand, the CAD/CAM milled coping group showed the highest axial internal gap, followed by the 3-D laser sintered group and the casting group. The values were 52.0 ± 8.9 µm, 74.3 ± 11.2 µm, and 55.8 ± 5.0 µm, for the casting, the CAD/CAM milled, and the laser sintered coping, respectively. The milled group showed significant differences to both the casting and laser sintered groups (*p* = 0.002), while the casting and laser sintered groups were not significantly different (*p* = 0.865). The occlusal internal gap value was 77.0 ± 21.0 µm in the casting group, 74.3 ± 18.2 µm in the CAD/CAM milled group, and 133.5 ± 22.8 µm in the laser sintered group. There were significant differences between all groups (*p* < 0.0001).

The correlation coefficient values (Table 3) illustrated that there are moderate correlations between the vertical marginal gap values and the internal gap width variables (*r* = 0.64), except for the weight of the silicone material.

## 3. Discussion

Optimal marginal adaptation is a major factor in the biological and mechanical stabilization of the fixed prosthesis. In this study, we compared the marginal accuracy and internal fit of Co-Cr alloy crown-copings, fabricated by casting, CAD/CAM milled, and 3-D laser sintered techniques. The data supports a rejection of the null hypothesis, as there were differences in the marginal and internal gaps among the three differently fabricated coping groups. However, the marginal and internal discrepancy of all groups was within the clinically acceptable range [28,42,43], of around 100 µm.

For the casting group in our study, we milled the castable pattern resin for the fabrication of the copings, in place of the conventional manual wax-up procedures. We tried to maintain the standardized design and the uniform thickness of the crown-copings, by eliminating the errors related to manual works. Also, the same captured data was used for the fabrications of all experimental crown-copings in our study. Therefore, it was possible to compare the marginal accuracy and the internal fit of the copings, focused on only the different metal fabrication methods.

Although both techniques of direct visualization under a microscope, and the internal gap measurements in the embedded and sectioned specimens, are well-established, most authors agree that these methodologies provide limited information [44,45,46], and it is impossible to use these methods in vivo. The replica technique and the weighting technique are convenient, reliable and valid, non-destructive ways to determine the clinical adaptation of restorations to tooth structure. Gonzalo et al. concluded that the shortcomings of a technique must be considered when interpreting results [45]. In this study, we used the direct visualization method, inspection after embedding and sectioning, and the weighting technique.

The descriptive terminology defining the “fit” varies considerably in previous studies. Moreover, the same term is used for different measurements, or different terms are used for the same measurement. No general guidelines exist on how to perform gap measurement restorations in vitro, or in vivo. Holmes et al. provided a critical approach to this problem [47], establishing several gap definitions according to the contour differences between the crown and tooth margin. However, in practice, it is almost impossible to describe a particular gap using only one definition, due to morphologic diversities, rounded margins, or defects [48]. This is one of the main reasons for a significant amount of variation, commonly reported among investigators. In the present study, we defined the marginal gap as the two-dimensional vertical marginal discrepancy measured from the coping, to the margin of the preparation. The internal gap is the vertical measurement from the internal surface of the coping, to the axial wall of the preparation. We divided the internal gap into three types, according to the measuring area.

To standardize the measurement, a standardized fabrication of the copings ensured a uniform thickness, and each specimen was sectioned at the same position to coincide with the reference indentations of the abutments. Additionally, the fitting surfaces of the copings were not refined, because the amount of refinement is hard to quantify or standardize. In other in vitro studies of marginal adaptation [39,49], preparation angles varied between 6° and 15°. In this study, all groups had the same 6° taper angle, to avoid being considered as a variable affecting marginal adaptation between groups. Since the machining tolerance of stock abutments is reported as approximately being in the range of ±0.01–0.1 µm, according to the manufacturers, the possible errors relating to the adaptation between the different abutments and the copings could be disregarded. We obtained the measurement data by positioning the specimens under the microscope using a special clamp. In this way, we could observe the marginal area of the abutment and coping junction from a directly perpendicular perspective. Moreover, misfit was assessed in five equidistant point areas per image, to reduce the operator bias. The random assignment of the abutments to the experimental groups, as well as the control of the individual human factors, can contribute to the validation of the findings.

In this study, the mean two-dimensional vertical marginal gap of the three groups was in the range of 38–73 µm, which were within the clinically acceptable range of 39–120 µm [31,32]. The cast coping group showed significantly smaller vertical marginal gap values than the CAD/CAM milled and 3-D laser sintered groups, and this finding is consistent with the results of previous studies [21,33]. This may be explained by the hard material, the Co-Cr alloy block, of the milled group, which is more difficult to cut precisely due to its hardness. More vibration and resistance of the milling axis during preparation could affect the accuracy of the milling procedure. Furthermore, the castable resin pattern of this study, used in casting specimens instead of the wax pattern, might attribute to the smaller marginal gap values of the casting group than is reported in other studies [29,36].

Variation in the internal fit can create stress concentrations, which may reduce the restoration strength [36]. In this study, the 3-D laser sintered group showed the largest average internal gaps, when compared to the other two fabrication methods. In contrast, Ucar et al. reported no significant differences between laser-sintered and cast Co-Cr sectioned crowns, when focusing on the internal gap [15]. Ortorp et al. reported that the laser sintered Co-Cr group showed lower discrepancies than the casting Co-Cr group, for the conventional fixed restorations [34]. However, we noted that these studies made no mention of the complete seating of the copings. In our study, we did not perform additional internal adjustments of all copings, except for the elimination of casting nodules under a microscope. Witkowski et al. evaluated the quality of the accuracy of copings after casting and machining, in before and after manual refinement, and concluded that internal refinement significantly improved the marginal accuracy [35]. These results explain the possible internal interference of the copings. Therefore, we considered a removal of the casting nodule, which inherently causes interference by voids in the casting procedure, unlike in the milling or laser sintering method.

The internal gap of the copings in all three groups was greater than those of the designed cement space in this study. This is different to the findings of other studies, which reported that the internal gaps of copings were almost the same as those of the designed cement space (30 µm). The gap size is affected by the thickness of the dental cement layer, influencing the seating of the restoration. Many factors affect film thickness, including preparation margin design, marginal configuration, surface roughness, cementation pressure, duration of cementation, powder/liquid ratio of the cement, types of cement, die spacers, and cementation techniques [42]. In this study, a recognized common feature in all three groups was a significantly greater occlusal internal gap than the axial and marginal values. This result is in agreement with previous studies [26,33]. We assumed that this significant discrepancy, particularly in laser sintered and milled groups, could be attributed to the process errors relating to the intrinsic setting of different tool path software programs used in manufacturing procedures. The vertical marginal gap and the internal gaps of the milled group, appeared greater than the casting group in this study. This can be explained by the two possible factors related to the fit of restorations produced by CAD/CAM milling system; the skill of the technician and the accuracy of the scanning process [17]. Other sources of error include the wear of milling instruments during milling and a change in the radius of the instruments during the milling procedure, which can reduce the milling precision [50]. A change of the milling instruments at regular intervals is highly recommended in order to control this factor [51].

We used a relatively new EOSINT M270 (EOS GmbH—Electro-Optical Systems, Krailling, Germany) laser sintering system, in addition to the popular and easily available EOS Cobalt ChRome SP2 granule^®^ (Biomain AB, Helsingborg, Sweden) Co-Cr alloy powder. Therefore, our study could contribute to the clinical relevance of the methods. However, there were some limitations in this study. We seated copings on the master abutments using finger pressure. This process is complicated when using a standardized tool, because the luting materials begin to set after a very short amount of time. Although this method clinically simulated the cementation of fixed restorations, which has been used in many other studies [27,37,38,39,44,45,46], and was performed randomly for all specimens by the same operator in order to keep the pressure consistent, we need to address the fact that the finger pressure was not fixed and quantified. If we design and use a new customized clamp device with load cells in the immobilization or adaptation of the abutment and coping, we could quantify or standardize the amount of force applied by the clamp screw in further study. The copings were not veneered in our study, but this may have presented another variable that could impact the marginal accuracy. Zeng et al. reported that repeated firing had no significant influence on the marginal accuracy of copings [52]. However, veneering can enlarge the gap size [53] and can induce a different change in the mechanical and physical properties, according to different metal compositions among experimental groups. In the present investigation, we initially focused on the coping without veneering.

Future research should include investigations into the marginal accuracy and internal gap of porcelain firing, besides single metal copings and multiple units of fixed dental prostheses. The composition of the Co-Cr alloy when using the laser sintered technique has a lower molybdenum content. The Co-Cr-M and Co-Cr-M-W alloy showed a remarkable increase in hardness after thermal treatment, by producing a homogeneous microstructure comprised of an intricate network [54,55]. There are some studies focusing on comparisons between corrosion behavior, cytotoxicity, and bond strength, and veneering porcelain, between different, commercially available Co-Cr alloys. However, few studies have compared the fit of restorations between different Co-Cr alloy brands. Further analysis is needed, comparing the fitness before and after veneering, according to the different commercial brands of Co-Cr alloys and laser systems.

## 4. Materials and Methods

### 4.1. Material and Preparation of Specimens

**Figure 2:**
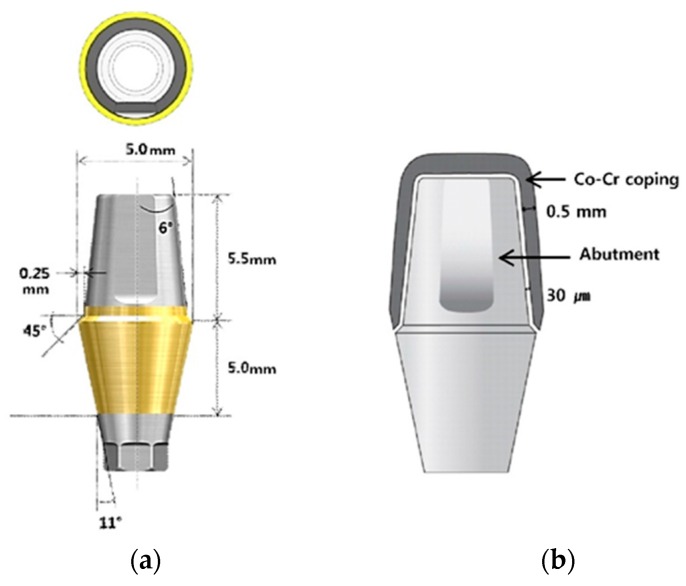
(**a**) The cross-sectioned image and sizes of the abutment used in this study; (**b**) Schematic diagram of the fabricated Co-Cr crown-coping. The thickness of the coping was designed to be 0.5 mm, and the cement gap was set at 30 µm.

A standard titanium implant abutment (Transfer type abutment, TS system, Osstem, Seoul, Korea) was used to produce the superstructures. It represents a mandibular first premolar with a beveled shoulder finish line, 6-degree taper angle, 5.0 mm diameter, hex, 5.0 mm gingival height, and 5.5 mm vertical height. Figure 2 shows the cross-sectioned image and sizes of the abutment used in this study, and the schematic diagram of fabricated Co-Cr crown-coping. The thickness of the coping was designed to be 0.5 mm, and the cement gap was set at 30 µm. The implant abutment was screwed onto a titanium implant replica (Lab analog, Osstem, Seoul, Korea), using the recommended torque (25 Ncm).

**Figure 3:**
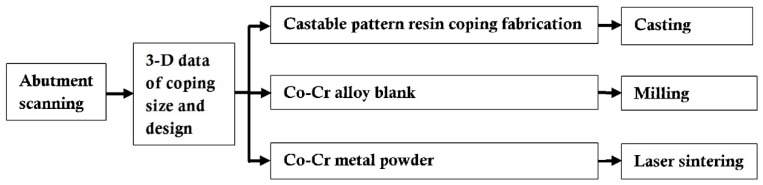
Workflow of the specimen preparation according to the fabrication methods.

Figure 3 shows the workflow of the fabrication stages of the specimen, according to the manufacturer’s recommendation and instructions for the three different methods. We fabricated 36 cobalt-chromium (Co-Cr) alloy copings; 12 copings for casting, 12 copings for CAD/CAM milled technology, and 12 copings for laser sintered technology.

Table 4 provides the alloy composition of each experimental group. For the fabrication of the crown-copings, the data of the coping size and design was captured using software (3Shape D800, 3Shape A/S, Copenhagen, Denmark). We fabricated the cast copings with castable pattern resin, using a three-dimensional system (ProJet^®^ 3510 MP, 3D Systems, Rock Hill, SC, USA). These castable resin patterns were invested in a phosphate-bonded investment material (UNI VEST NON-PRECIOUS, SHOFU Inc., Kyoto, Japan) with metal ring, and cast with the Co-Cr-based metal alloy (JEWOOS02, JEWOO M-Tech, Seoul, Korea). Casting is usually carried out by induction heating, in combination with centrifugal casting (Casting machine, Seki Dental Co., Seoul, Korea). The cooling procedure, deflasking, and blasting with 250 µm aluminum oxide, at a pressure of 3 bar and a 20 mm distance between the nozzle and specimen surface, with an angle of 45°, were all carried out. The casting sprues were cut with a separating disc (0.6 mm, No. 43135, Orbis Dental, Offenbach, Germany). The casting beads on the inside of the copings were removed with rotating instruments (No. H71EF, Brasseler GmbH dn Co., Lemgo, Germany). The thickness of the copings was confirmed with a thickness gauge (Iwanson crown wax caliper, SurgiDental instruments, Fort Worth, TX, USA). The margin and the internal casting beads were examined with a stereomicroscope (Wild M1B, Leica Geosystems AG, Heerbrugg, Switzerland) at ×14 magnification. No additional internal adjustment of the copings was performed, except for the elimination of casting nodules with rotating instruments.

The 3Shape CAD data of coping was also sent to a communicating 5-axis milling machine (DNM-500, SMT Solution Co., Seoul, Korea) for the fabrication of the CAD/CAM milled copings, from the Co-Cr alloy blanks (Starbond CoS, S&S Scheftner GmbH, Mainz, Germany). The copings were milled by the machine to the wall thickness, as defined by the computer. The size of the smallest milling bur was 0.8 mm. We prepared the laser sintered specimens from Co-Cr powder (particle size of 15 µm), using direct metal laser sintering (DMLS) technology. The EOS Cobalt ChRome SP2^®^ granule (Biomain AB, Helsingborg, Sweden) was used. The same 3Shape CAD data of coping was sent to the production center (E-Master Dental Hub, Seoul, Korea), where the laser sintering was to be performed using the direct metal laser sintering system (EOSINT M270, EOS GmbH—Electro-Optical Systems, Krailling, Germany). The laser scan speed and layer thickness were fixed at 7.0 m/s and 30 µm, respectively. The copings were fabricated under a laser power of 200 W and scan spacing ranged from 0.1 to 0.2 mm, and was sandblasted with 250 µm aluminum oxide at a pressure of 3 bar, before the heat treatment. The heat treatment was performed in a furnace (LAB24 SF-25, Dongseo Science Co. Ltd., Seoul, Korea) at 800 °C for 5 h, for releasing residual internal stress. No treatment after CAD/CAM milled copings and laser sintered copings fabrication was performed.

### 4.2. Fit Evaluations and Statistical Analysis

We distinguished all copings of each group (*n* = 12) by their assigned numbers. We adapted each coping on the abutment, intermediated with silicone pressure indicator material (Fit Checker II, GC Corporation, Tokyo, Japan). After mixing equal amounts of base and catalyst, we placed the silicone material inside each coping. Following this, we seated copings on the abutment using finger pressure [44], simulating the clinical application of a luting agent. Following the removal of excess unpolymerized silicone material at the margin, finger pressure was further applied for one minute. After polymerization of the silicone material, we removed copings from the abutment and weighed the silicone using an analytical balance (OHAUS PA214 Pioneer™, OHAUS Co., Parsippany, NJ, USA). All measurements were performed by the same operator and repeated three times. The order of measurements within three groups was randomized, using a random number generator (Microsoft Office Excel 2010, Microsoft Co., Redmond, WA, USA).

**Figure 4:**
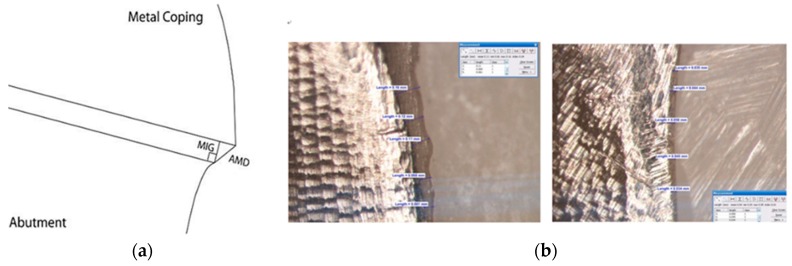
Schematic sectioned view of reference points for evaluation (**a**) and measurement images (**b**) the marginal fit in this study. Marginal internal gap (MIG); the perpendicular measurement from the internal surface of the coping to the axial wall of the abutment at the end of the margin, Absolute marginal discrepancy (AMD); the angular combination of the marginal gap and the extension error which is measured from the margin of the coping to the cavosurface angle of the abutment. In this study, we measured the AMD as the two-dimensional vertical marginal gap. (5 points × 4 areas × 12 specimens × 3 groups, total 720 points).

The two-dimensional vertical marginal discrepancy was assessed by measuring the distance between the margins of the copings and their respective abutments at five points per image, at four predetermined equidistant points (Figure 4). We utilized a stereoscopic zoom microscope (SZM-45T2, Sunny Optical Technology Co., Yuyao, China) at ×40 magnification. For these measurements, the copings were sequentially placed on the master abutment and immobilized by acustomized clamp with a predetermined screw stop and frame. The abutments were fitted in a special support to situate the vertical gap perpendicularly to the optic axis of the stereomicroscope, thus guaranteeing repeatable projection angles. We marked the four equidistant points on the submarginal surface of the abutment, before the coping adaptation procedure. We took digital photographs of the four-points area of the abutment per coping, using a digital SLR camera (Nikon D50, Nikon Inc., Melville, NY, USA) attached to the stereomicroscope with a millimeter ruler. This millimeter ruler, at the same magnification, was used as a standardized reference in the calibration of the measurement software (Image J 1.44p, National Institute of Mental Health, ‎Bethesda, MD, USA). The camera reproduced a ×40 magnification on a high-resolution computer monitor, so that an image of the marginal discrepancy could be examined using software. The software determined the mean separation between the margin of the coping and the abutment line in micrometers. To ensure that the software was correctly calibrated for the data collection, we preceded a measurement of a known distance (0.5 mm) at every point, using the image of the millimeter ruler. We performed the evaluation of the mean vertical marginal gaps (calculated by five points per image, four images per coping, 12 copings per group, producing a total of 720 measurements) according to the literature [56,57], as well as by considering the average maximum marginal gap within one group.

**Figure 5:**
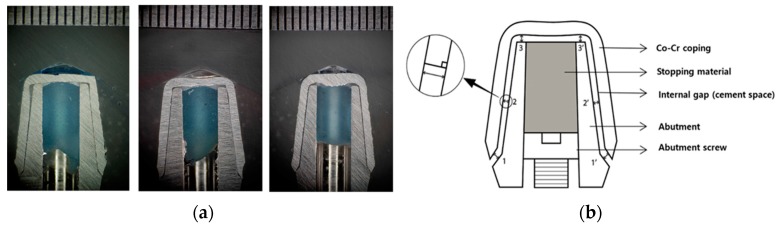
(**a**) Specimens for internal gap measurements in the casting, CAD/CAM milled, and laser sintered groups; (**b**) Schematic view of six standardized measurement areas for internal gap: two marginal point areas (1, 1’), two axial point area (2, 2’), and two occlusal point area (3, 3’). Measurement location of the marginal gaps (1, 1’) was the center of chamfer-area, and the measurement location of axial gaps (2, 2’) was the center of the axial wall, starting at the end-point of the margin and continuing until the transition point with the occlusal area. Measurement location of occlusal gaps (3, 3’) included the center of the occlusal surface of the coping, on both sides of the access hole (5 points × 2 locations × 3 areas × 12 specimens/group). A small circle on the left side shows the internal gap measurements as the perpendicular distance between an outer surface of the abutment and the inner surface of the coping.

After vertical marginal gap measurement, we cemented each coping to the abutment, which was screwed onto a titanium implant replica (Lab analog, Osstem, Seoul, Korea) with the recommended torque (25 Ncm), using resin-modified glass ionomer cement (FujiCEM™ 2, GC Corporation, Tokyo, Japan). Then, we applied firm finger pressure for five minutes, until the hydraulic pressure was relieved. Furthermore, we removed the excess cement after polymerization. All specimens were embedded in self-curing acrylic resin (Ortho-Jet™, Lang Dental Manufacturing Co. Inc., Wheeling, IL, USA) in the center of the prefabricated plastic mold. Each block was sectioned longitudinally in the labiolingual direction, using an electronically controlled diamond saw (KDMT-285, Kyungdo Precision Co. Ltd., Seoul, Korea). Sectioned surfaces of each specimen were polished with a series of silicon carbide (SiC) abrasive papers (160, 320, and 800 grit) to remove the metal particles that were adhered to the surfaces using a grinder-polisher machine (KDMT-300, Kyungdo Precision Co. Ltd., Seoul, Korea). Following this, the sectioned surfaces were ultrasonically cleaned in water (WiseClean^®^WUC, DAIHAN Co., Seoul, Korea) for five minutes in order to remove the surface contaminants. The order of the experiments within the three groups was randomized using a random number generator, as previously described, for each of the cementation, sectioning, and polishing procedures, in order to eliminate any bias that might affect the results. After initially obtaining photographs of each cross-sectioned specimen with a stereomicroscope (SZM-45T2, Sunny Optical Technology Co., Yuyao, China) at ×40 magnification, three digital images were made for each specimen, using a digital SLR camera (Nikon D50, Nikon Inc., Melville, NY, USA) attached to a stereomicroscope. Photographs were produced with a digital camera (Nikon D50, Nikon Inc., Melville, NY, USA) and transferred to the imaging data program (Image J 1.44p, National Institute of Mental Health, Bethesda, MD, USA). The measurements of the internal gap in this study were divided into three different areas of interest for better comparisons, according to the terminology reported by Holmes et al. [47]. The internal gap width was measured for three points at six standardized zones, shown in Figure 5. The marginal zone was the center of the beveled shoulder area, and the axial zone was the center of the axial wall, starting at the end-point of the margin and continuing until the transition point with the occlusal area. The occlusal zone included the center of the occlusal surface of the coping, on both sides of the access hole. We measured each point three times using a single investigator, and determined the mean value.

Statistical comparisons of the weight of the silicone material, two-dimensional vertical marginal gap, and internal gap for the three groups of Co-Cr alloy copings, were performed using one-way ANOVA. The Scheffe’s test was performed to determine the significant differences between groups, and the level of significance was set at *p* = 0.05, while calculations were handled by the statistics software package (SPSS 19.0, IBM Co., Armonk, NY, USA). Also, the Pearson’s correlation analysis was used to assess the existence of the interrelation between the methods used in this study for fit evaluation.

## 5. Conclusions

Laser sintered technology has been introduced in clinical use and can be utilized more widely, accompanied by the digitalization of dentistry and the development of direct oral scanning devices. Also, laser sintering technology has an advantage relating to the minimization of human error during the manufacturing procedures, maintaining a consistent quality of restorations. Furthermore, the construction costs of prostheses might be reduced through large-scale production at one time. In this study, the marginal adaptability of Co-Cr alloy copings fabricated by a 3-D laser sintered technique was worse than that of copings fabricated by the casting and milled methods. However, all misfit values in this study could be considered clinically acceptable, since marginal discrepancies of up to 150 µm have been admitted for implant-cemented prostheses. Therefore, this new laser sintering system can compete with the conventional cast and CAD/CAM milled systems for clinical fit, and can achieve more accurate marginal and internal fittings with further improvements.

## Figures and Tables

**Table 1:** The silicone weight for the casting, CAD/CAM milled, and laser sintered groups.

Group	Mean (g)	SD
Casting	0.006 ^a^	0.001
CAD/CAM milled	0.007 ^b^	0.001
3-D laser sintered	0.007 ^b^	0.001

^a,b^ Different letters correspond to statistically differences for groups (*p* < 0.05).

**Table 2:** The vertical marginal gap for the casting, CAD/CAM milled, and 3-D laser sintered groups.

Group	Mean (µm)	SD
Casting	38.2 ^a^	6.2
CAD/CAM milled	51.5 ^b^	7.0
3-D laser sintered	72.5 ^c^	12.4

^a–c^ Different letters indicate that values are significantly different between groups (*p* < 0.05).

**Table 3:** Pearson’s correlation coefficient among weight, marginal gap, and internal fit.

Group	Weight	Marginal Gap	Average Internal Fit
Weight	1	-	-
Marginal Gap	0.257	1	-
Average Internal Fit	0.008	0.625	1

**Table 4:** The chemical composition of the casting, milled, and laser sintered Co-Cr alloys as a percentage, according to the manufacturer’s instructions (wt %).

Alloys	Co	Cr	Mo	W	Si	Fe	Mn
Casting	63	28	5.5	<3.5			
CAD/CAM Milled	59	25	3.5	9.5	1.0	<1.5	
3-D Lasersintered	63.8	24.7	5.1	5.4	1.0	<0.5	<0.1

All alloys are for the fabrication of crowns.
